# Bibliometric and Visual Analysis of Studies on Ceramic Membranes: A Review

**DOI:** 10.3390/membranes14070144

**Published:** 2024-06-25

**Authors:** Hao Xiong, Xianfu Chen, Jun Feng, Fan Zhang, Minghui Qiu, Qi Zhang, Yiqun Fan

**Affiliations:** 1 State Key Laboratory of Materials-Oriented Chemical Engineering, College of Chemical Engineering, Nanjing Tech University, Nanjing 211816, China; 2 Energy and Environmental Materials Research Department, Suzhou Laboratory, Suzhou 215123, China; 3 National Intellectual Property Information Service Center in Colleges and Universities, Nanjing Tech University, Nanjing 211816, China; 4 Ocean College, Zhejiang University, Zhoushan 316000, China; 5 Jiangsu Province Key Laboratory of Fine Petrochemical Engineering, School of Petrochemical Engineering, Changzhou University, Changzhou 213164, China

**Keywords:** ceramic membrane, bibliometric, visual analysis, VOSviewer, CiteSpace

## Abstract

As a high-performance separation material, the ceramic membrane has played a crucial role in addressing resource, energy, and environmental challenges. Here, we carried out literature retrieval and collection for the research of ceramic membranes based on the Web of Science. The retrieval strategy was quantitatively evaluated from two dimensions: recall and precision. The distributions of publication time, journal, and related subjects were systematically analyzed. With the help of CiteSpace and VOSviewer, the literature was visually analyzed through the co-occurrence map of authors and the cluster network of keywords. The findings indicate a strong correlation between ceramic membrane research and the field of Chemical Engineering. A core group of authors has emerged as prominent contributors in this area of study. Additionally, there is a notable long-tail effect observed in the application of ceramic membranes. Despite their current low-frequency usage and high-volume potential, these applications hold substantial promise for future scientific research and industrial development.

## 1. Introduction

Membrane technology plays a crucial role in addressing resource, energy, and environmental challenges [1,2,3]. Its effectiveness in removing pollutants, reducing resource consumption, and mitigating environmental impact makes it a valuable tool for creating a more sustainable future [4]. Ceramic membranes, crafted from inorganic substances like alumina, silica, zirconia, and titania, exhibit exceptional resistance to severe conditions, encompassing high temperatures, extreme pH levels, aggressive chemicals, and oxidizing agents [5,6]. Additionally, due to their hydrophilicity and narrow pore size distribution, ceramic membranes exhibit high permeability and separation accuracy, as well as excellent anti-fouling properties [7,8]. These properties have led to ceramic membranes gaining significant attention in water and wastewater treatment, air pollution control, and improving industrial process efficiency and stability [9,10].

To fully understand the current research status of ceramic membranes and explore research hot spots and frontiers, an in-depth analysis of the scholarly communication is necessary [11]. By employing bibliometric and visual analysis, researchers, policymakers, investors, industrial professionals, and other stakeholders can gain valuable insights into the intellectual structure, knowledge gaps, and opportunities in the field of ceramic membranes [12,13]. This understanding can inform decisions and strategies to accelerate the development of high-performance ceramic membranes and support a more sustainable future [14].

In the preliminary work, scholars employed bibliometric analysis as a method to systematically categorize the relevant literature [15,16]. This approach was applied to examine the advancements in membrane applications within environment fields, yielding promising outcomes [17,18,19]. Some noteworthy examples include an investigation into the current status and prospects of polyaniline membrane utilization in water treatment [20], an exploration of the potential for large-scale commercial implementation of membrane processes in paraffin/olefin separation systems [21], an evaluation of the progress in membrane distillation technology for seawater desalination [22], and an analysis of global characteristics and trends of research on ceramic membranes from 1998 to 2016 [23], etc.

Nonetheless, there is still a paucity of bibliometric and visual documentation concerning ceramic membranes. This review utilized the Web of Science database as the primary literature source. Extensive explorations and meticulous organization of ceramic membrane literature were carried out, covering dimensions such as distribution of publication time and regions, sources, authorship, keywords, and the identification of highly cited publications. Utilizing two efficient tools, VOSviewer and CiteSpace, a series of visual maps were generated. These visual maps were then employed to analyze the research hotspots and developments in the ceramic membrane field, aiming to bolster their application in environmental fields.

## 2. Retrieval Method

### 2.1. Retrieval Strategy

Extracting pertinent literature is essential in the analytical process. Ensuring high-quality sources requires a carefully crafted literature retrieval strategy. Precision and recall rates are key indicators of retrieval success [24]. Enhancing precision reduces the impact of irrelevant data (noise) on the analysis, whereas increasing recall ensures the thoroughness of the results. However, there is often a trade-off between these two indicators [25].

According to the insights of visual analytics expert Professor Chen [26], prioritizing a higher recall rate is a more efficient and safer approach than striving for an elevated precision rate without due consideration. Aligning with this perspective, this study adopts a keyword search method to strike a balance between high recall and precision when crafting the search formula [27]. The methodology for choosing subject terms primarily involves two stages: Initial searches with the keywords *ceramic membrane* facilitate a broad categorization of the literature and a breakdown of pertinent technology. Following this, high-frequency keywords are pinpointed and established as the central subject terms in the search formula.

In the specific research and application contexts of ceramic membranes, precise names such as *alumina membrane* or *titanium oxide membrane* are commonplace. To boost the recall rate, these frequently employed material names and their chemical formulas are included as additional keywords. Adhering to this strategic framework, the search fields are chosen to include the topic (TS) and abstract (AB), utilizing logical operators such as AND and OR to obtain the following formulate:

*(AB = membrane AND TS = (Perovskite OR ceramic OR aluminum oxide OR alumina OR corundum OR zirconia OR zirconium oxide OR zirconium dioxide OR zincite OR titania OR titanium oxide OR titanium dioxide OR silica OR silicon oxide OR yttria OR yttrium oxide OR hafnia OR hafnium oxide OR ferric oxide OR iron oxide OR aluminum nitride OR boron nitride OR boramon OR silicon nitride OR carborundum OR silicon carbide OR carbofuran OR nicalon OR titanium carbide OR mullite OR cordierite OR silicon OR kaolin OR bentonite OR bauxite OR attapulgite OR portland OR apatite OR quartz OR flay ash OR clay OR Al_2_O_3_ OR TiO_2_ OR ZrO_2_ OR SiO_2_ OR Y_2_O_3_ OR HfO_2_ OR Fe_2_O_3_ OR AlN OR BN OR Si_3_N_4_ OR SiC OR TiC OR MXene))*

Considering the initial search results produced an abundance of less pertinent literature, a significant impact from noise is likely. Consequently, implementing a series of refinement strategies is crucial to improve the relevance of the retrieved results. The approach to refinement is as follows:

(1) Upon reviewing the initial results, certain terms such as *cell membrane*, *Nafion*, *membrane proteins*, *biomembrane*, and *proton exchange membrane* were identified as significant sources of interference. These terms have consequently been excluded to improve the specificity of the search.
(2) An analysis of the search outcomes also revealed the inclusion of literature from unrelated subject categories, such as virology, surgery, and zoology. These categories have been systematically excluded through the subject category classification feature provided by Web of Science (WOS).

Implementing these refined strategies should significantly reduce irrelevant entries and hone in on the most pertinent literature, thus optimizing the search process for better-quality results.

### 2.2. Evaluation and Analysis Methods

The process for evaluating the recall rate is outlined as follows: The top five institutions from the search results are subjected to an institution-specific search within the WOS literature database. Through careful manual screening, non-relevant and low-correlation literature is eliminated, culminating in the identification of the number of articles (*N*_A_) for the five institutions. Further refinement involves additional searches and manual exclusion of low-correlation literature to ascertain the number of pertinent articles (*N*_P_) for the five institutions. The recall rate (*r*, %) is then calculated using Equation (1):

(1) $$r=\frac{N_{P}}{N_{A}}\times 100$$

For the precision rate, the approach entails a random selection of 300 articles from the results. High-relevance articles are identified through manual review, and the number of articles is counted as *N*_B_. Precision (*p*, %) is calculated using Equation (2):

(2) $$p=\frac{N_{B}}{300}\times 100$$

The determination of core authors is based on an analysis of publications: Price examines the correlation between the number of scientists and the amount of scientific literature, noting the enormous role of elites in the development of science, as well as the quantitative relationship between scientists at different levels of competence. Many studies identifying core authors are based on Price’s law. The author with the highest number of publications (*n*_max_) is first identified, followed by setting a threshold for core author publications based on Price’s law [12]. The minimum number of publications (*m*) for a core author is then calculated using Equation (3):

(3) $$m=0.749\times \sqrt{n_{max}}$$

In analyzing literature retrieval outcomes, two software are employed: VOSviewer and CiteSpace, each with unique benefits [28,29]. The analysis process of the software is mainly divided into three steps: data standardization processing, cluster analysis, and visualization. First of all, when standardizing, the characteristic information in the literature, such as the year of publication, author, keywords, etc., will be analyzed to obtain the correlation between the data. Then, the two software will use the method of cluster analysis to reveal the association and group the data objects into multiple classes or clusters, and the principle of division is that the objects in the same cluster have good similarity, and the objects between different clusters are quite different. Finally, these results are presented in the form of a map to obtain a visualization result.

VOSviewer, created by Nees Jan van Eck and Ludo Waltman of the Netherlands, specializes in forming bibliometric networks. CiteSpace, developed by Professor Chaomei Chen from Drexel University, is proficient in dissecting literature’s key features and producing visual network maps. In this research, VOSviewer [30] (version: 1.6.16) and CiteSpace (version: 6.2.R2) were applied to examine the literature retrieval findings, establish bibliometric networks, and create visual maps for clearer interpretation.

## 3. Result and Discussion

### 3.1. Evaluation of Retrieval Strategy

The Web of Science Core Collection was applied as the primary database for the literature search via the professional search mode. The search span covered records from 1 January 1990 until 30 December 2022, with a focus on journal literature. Initially, approximately 18,500 potential publications were identified, excluding reviews and conference proceedings. Further refinement was undertaken to minimize the presence of irrelevant disciplines, such as neuroscience, drug delivery, and various humanities, yielding around 15,250 pertinent publications.

Firstly, the recall rate was assessed. Based on the search findings, the leading five institutions were pinpointed. Using Equation (1), the number of publications associated with each institution is detailed in Table 1. The recall rate was calculated to be 84.20%. Secondly, the precision rate was evaluated. Out of 300 papers randomly selected from the search outcomes, 77 were deemed less relevant to the field of ceramic membranes, resulting in an *N*_B_ value of 223. The precision rate was calculated to be 74.33% by Equation (2). Overall, the specialized search strategy devised by the authors has yielded high recall and precision rates, thereby effectively ensuring the completeness and dependability of the analysis that follows.

**Figure 1:**
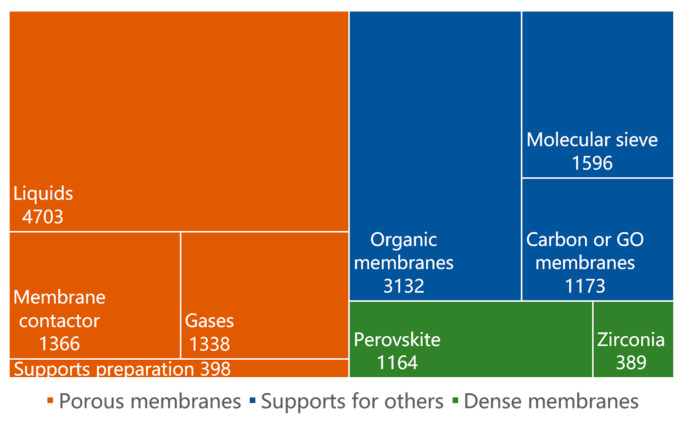
Literature classification and proportion statistics.

Upon evaluating the search strategy and its outcomes, 15,250 relevant publications were identified and organized. The literature is divided into three categories according to the colors, all the literature in the orange parts is related to porous ceramic membranes, and the literature in the green parts is related to dense ceramic membranes. It is worth noting that porous ceramic membranes are also commonly used as supports for other membranes like zeolite membranes, carbon membranes, graphene oxide (GO) membranes, and various organic membranes as depicted in the blue parts (Figure 1). Approximately 38.64% of the publications discussed porous ceramic membranes serving as supports for other membranes. This is owing to their robust mechanical properties, resistance to high temperatures, and resilience to acids and alkalis. Additionally, the statistical data suggest that the predominant application of porous ceramic membranes lies within liquid systems, accounting for more than 60% of their applications. There are 1553 publications on dense ceramic membranes, mainly including perovskite and zirconia-based membranes. Compared with porous ceramic membranes, the total number of publications on dense ceramic membranes accounts for less, only 10.18%.

### 3.2. The Spatial and Temporal Distribution of the Literature

**Figure 2:**
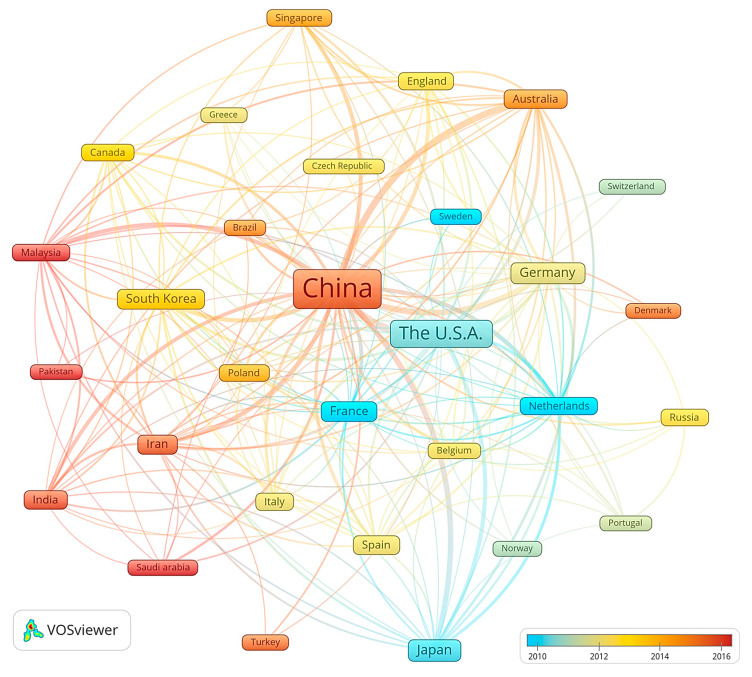
Regional distribution of literature.

The top 30 geographical areas with the highest publication output were selected, and a visual map was created to illustrate the regional distribution of the literature as shown in Figure 2. Each node on the map signifies a country, with the size correlating to the number of publications from that area. The links between nodes denote collaborative efforts, with the thickness indicating the extent of collaboration. The numerical scale of the color bar represents the trend over time. The node color transitioning from cool to warm tones represents the chronological spread of publications.

The People’s Republic of China, the United States of America, Japan, Germany, and South Korea emerge as the top five regions by publication number. In terms of inter-regional collaboration, prominent publication areas are seen to engage with other regions. The strongest collaborative ties are observed among the People’s Republic of China, the United States of America, and Germany. Among the 30 nodes represented on the map, the average number of connections per region is 28.9, signifying a widespread prevalence of cross-regional collaboration. This highlights a strong synergy among key areas engaged in ceramic membrane research. However, the intensity of cooperation varies, likely influenced by the number of publications from different regions.

When examining the temporal distribution of publications in ceramic membrane research, distinct regional variations become apparent. The chromatic distinction of nodes on the map reveals that the literature outputs from regions like the People’s Republic of China and India are discernibly warmer in color, signifying a surge in more recent publications. Conversely, nodes representing the United States of America, Japan, and France exhibit cooler hues, suggesting a substantial contribution to earlier research in the field. Regions such as Saudi Arabia, Malaysia, Turkey, and Denmark, despite having fewer publications to date, show warmer node colors and are linked with strong collaborative ties. This suggests burgeoning growth and future potential in ceramic membrane research within these areas.

**Figure 3:**
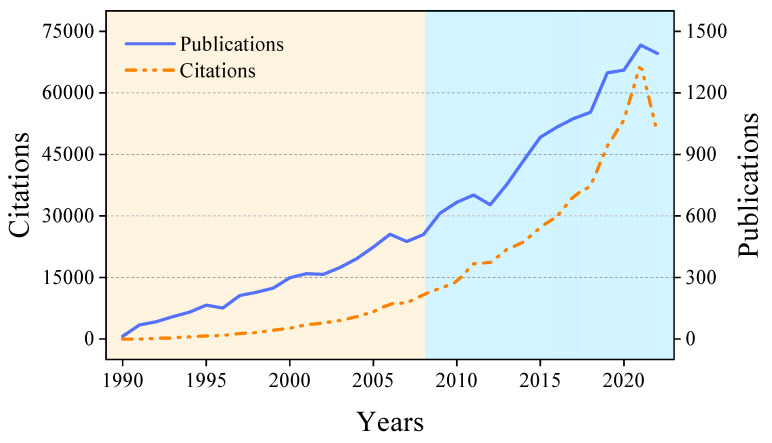
Annual publications about ceramic membranes from WOS.

The historical data of publications and citations are visualized in Figure 3. The publication trend in ceramic membrane research can be segmented into two stages. The initial phase is indicated in the yellow part, spanning 1990–2008, and is marked by a steady but modest annual increase in publications, averaging approximately 264.6 publications per year and culminating in nearly 5028 publications. The subsequent phase is indicated by the blue part, from 2009–2021, experienced a pronounced surge in publication activity, with an average of 961.8 publications annually, amounting to 12,504 publications during this period. This suggests a sustained upward trend in future research. Notably, there were 1392 publications in 2022, slightly less than the previous year, likely due to the considerable impediments the global COVID-19 pandemic has imposed on scientific endeavors worldwide [31].

Since 2009, the citations have shown a rapid growth trend (Figure 3). By 2021, citations surged to 67,082, a substantial increase of approximately 25.49% from the previous year. It is also significant to note the increasing ratio of publications to citations. As detailed in Table 2, in 1992, the average ratio of citation to publication was less than 2. By 2020, this ratio had soared beyond 40. This phenomenon is primarily driven by two factors. First, the accumulation of earlier publications has expanded the range of research topics, resulting in more frequent referencing within the field. Second, the growing recognition of ceramic membranes significantly increased their citations in other fields involving membrane applications.

In light of the publications and collaborative dynamics previously discussed, it is evident that contributions from the People’s Republic of China and its international research collaborations occupy a pivotal role. To investigate the impact of different literature databases and the influence of journals in different languages on publication outputs [32], the number of publications within the field of ceramic membranes was analyzed using the China National Knowledge Infrastructure database (CNKI).

As a comprehensive repository of Chinese scholarly works, CNKI provides a lens into the progress of ceramic membrane research in China. Employing search strategies analogous to those described in Section 2.1 and adhering to CNKI’s syntactic criteria, the number of publications from CNKI and patents in China was collected. Additionally, information on some important scientific research projects in China can be obtained from the Big Data Knowledge Management Service Portal of the National Natural Science Foundation of China (NSFC) as shown in Table 3.

**Figure 4:**
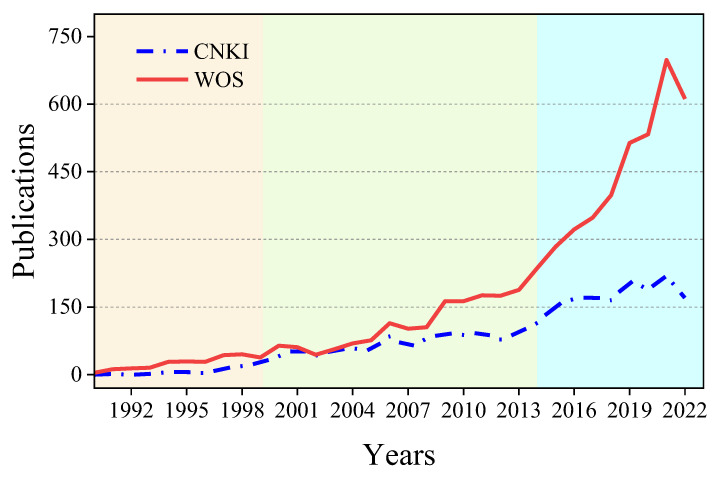
Comparison of publications from CNKI and WOS databases.

The annual publications were compared with those from WOS as shown in Figure 4. The year-on-year change trend in the publications from CNKI is basically consistent with WOS. The trajectory of ceramic membrane research in China can be segmented into three distinct phases:

(1) The initial period (1990–1999) is indicated in the yellow part, marks the nascent stage of development. Beginning in the late 1980s, the National Natural Science Foundation of China launched projects focused on inorganic membrane research. This initiative spurred major scientific institutions such as the Chinese Academy of Sciences, the University of Science and Technology of China, and Nanjing Tech University to commence their related research efforts.
(2) The second period (2000–2014) indicated in the green part, saw a consistent increase in publications. This era of rapid development and industrialization of ceramic membrane technology can be attributed to robust national science and technology projects and the burgeoning membrane separation market in China. Nanjing Tech University pioneered the mass production of tubular ceramic membranes domestically. During this time, significant advancements were made in ceramic membrane research, spanning from theoretical foundations to practical applications.
(3) The current phase, (2014 to the present) indicated by the blue part, is characterized by stabilization. This could be due to two primary factors: the trend towards internationalization, with researchers favoring publications in international journals, and a shift driven by national policies on scientific and technological achievement conversion and market forces, leading some researchers to focus on industrialization, with the outcomes of the research being published in patents and other forms [33,34]. This has resulted in a slowed growth trend in academic publications. From 2014 to 2022, the annual publication rate stabilized at around 170 papers.

Both CNKI and WOS databases experienced an uptick in publication numbers from 2008 to 2010, marked by escalating growth rates. However, after 2015, their paths diverged: WOS reported a continual surge in Chinese publications, whereas CNKI’s growth started to plateau. Delving into WOS data from the last 15 years reveals a consistent increase in annual publications from the People’s Republic of China, accounting for over one-third of all records since 2018. This variance suggests that while CNKI’s publications began to stabilize, there was a significant rise in Chinese research in the ceramic membrane field noted by WOS, indicating a shift toward publishing in international journals.

According to Professor Shneider’s four-stage model of scientific research field development [35], the progression of ceramic membrane research as indicated by publication numbers corresponds well with the model’s initial three stages. Presently, ceramic membrane research seems to be in the third stage. Owing to their exceptional separation properties and durability in various settings, ceramic membranes are finding broader use in numerous domains such as petrochemicals, biomedicine [36,37], metallurgy, energy, and environmental sciences. The expanding range of these applications highlights an increasing convergence with multiple disciplines, mirroring the research evolution as outlined in the third stage of Shneider’s four-stage model.

### 3.3. Distribution of the Literature Based on the Source of Publication

Upon analyzing the subject categories of journals according to the Journal Citation Reports (JCR) discipline classification, the top ten journals were identified and are presented in Table 4. Collectively, these ten journals contribute about 5515 publications, representing 29.81% of the total. Expanding the analysis to include the top 50 journals brings this total to approximately 9910, accounting for 53.57% of all publications (Table S1). This indicates a relatively broad distribution of publications across various journals. Notably, journals within the Chemical Engineering and Water Resources categories tend to have a higher number of publications. This observation, combined with the application system distribution mentioned in Section 3.1, underscores the critical role of ceramic membranes as advanced separation materials, especially in liquid systems, like their typical use in petrochemicals and water treatment.

**Figure 5:**
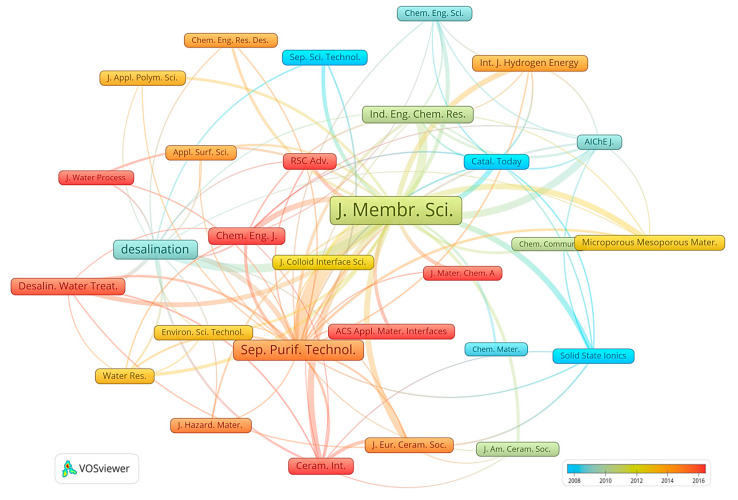
Co-occurrence map of the top journals in the area of ceramic membranes.

Furthermore, an analysis of citations between journals was conducted using a visual representation (Figure 5). In this map, the size of each label represents the number of citations a journal has received, and the thickness of the lines connecting the journals indicates the volume of cross-citations among them. The numerical scale of the color bar represents the trend over time. The total link strength displayed in this visualization serves as a measure of the extent to which a journal is cited by other publications, providing insights into its influence and interconnectedness in the research of ceramic membranes.

Citations serve as a crucial metric for gauging a journal’s influence, whereas the total link strength value offers deeper insight into the journal’s standing within the specific field. According to Figure 5, there is a notable correlation between journals that have high citations and publications. For example, the journals *Journal of Membrane Science*, *Separation and Purification Technology*, and *Desalination* have total link strength values that closely align with their number of publications. However, it is worth mentioning that some journals publish fewer papers but have more cross-citations with other journals. For example, *AIChE Journal* and *Journal of European Ceramic Society*, although they do not appear in the top ten list of publications, their total link strength is high. In addition, considering the aspect of publishing time, journals like *Ceramics International* and *Chemical Engineering Journal* have recorded a high number of publications and citations in recent years. This indicates a significant level of current activity and interest in the field of ceramic membranes, reflecting their growing importance and the ongoing, dynamic research within this area.

### 3.4. Analysis of Author Cooperation

Examining the central group of authors and their collaborative networks offers valuable insights into the leading scholars and primary research entities in the ceramic membrane field. By tallying the publications of authors, the highest number of publications (*N*_max_) identified in this domain is 162. Utilizing Equation (3), the threshold for the minimum number of publications (*m*) to be considered a core author in ceramic membrane research is computed to be approximately 9.53. Thus, the publications by core authors in ceramic membranes are expected to be over 10. A research field is deemed to have a core author group when the ratio of publications by core authors to the total publications exceeds 50%. According to the WOS search findings, 658 authors meet this criterion, being collectively responsible for about 12,270 publications. This represents 66.32% of the total publications in the ceramic membrane field, signifying the establishment of a core author group.

**Figure 6:**
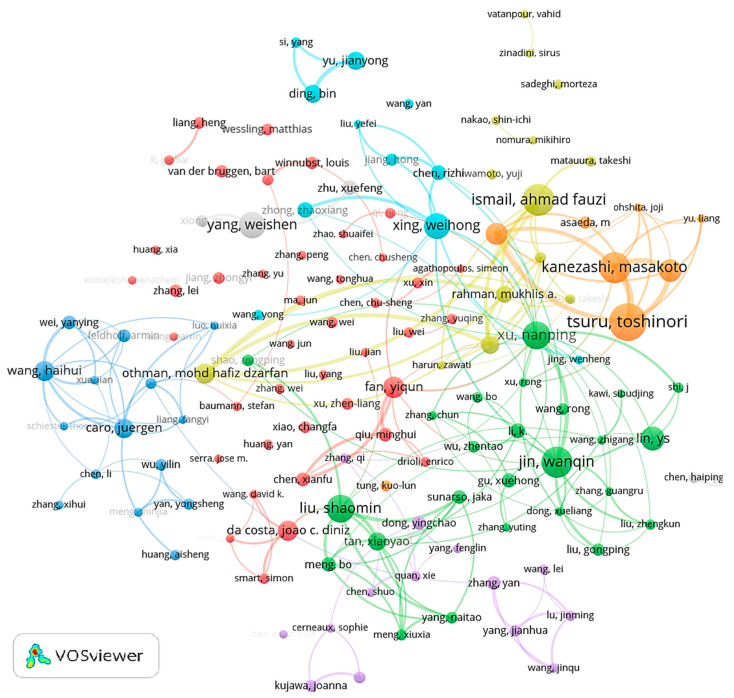
Co-occurrence map of the top 200 core authors in the area of ceramic membranes.

The co-occurrence map of the top 200 core authors is illustrated in Figure 6. Each node represents an author, with the size of the node indicating the number of publications by the author. The links between nodes signify collaborative relationships, with the thickness of the lines indicating the strength of the connections, and the colors representing different cluster groups. The map shows a high degree of interconnectivity within clusters, with authors who have a large number of publications forming distinct networks. Collaboration is particularly strong at the core of these groups, though the connections extending outward from these central clusters are less pronounced.

In general, an author’s contribution to the field can be reflected in the volume of publications and connections with adjacent author nodes. Typical authors are Toshinori Tsuru, Juergen Caro, Weihong Xing, Shaomin Liu et al. Professor Toshinori Tsuru has done a lot of work on the preparation, application, and separation mechanism of silicon-containing membrane materials such as silica membranes, silicon carbide membranes, silicone membranes, etc., especially in the study of the performance of membranes in permeation processes such as pervaporation and reverse osmosis [38,39]. Professor Juergen Caro’s experience in the field of gas separation membranes is mainly in supported zeolite, metal-organic framework (MOF), and covalent organic framework (COF) membranes. In polymer matrices, scale-up-friendly hybrid matrix membranes filled with MOF or COF nanoparticles are also processed into gas separation membranes [40,41]. Professor Weihong Xing is committed to the design, preparation, and application of high-performance membrane materials and has made research progress in membrane reactors, membrane water treatment technology, and air pollution control technology around the application needs of energy conservation and emission reduction in the chemical industry [42,43,44]. Professor Shaomin Liu is mainly engaged in the research and development of inorganic membrane materials and membrane modules, the mechanism of membrane reactor separation and mass transfer, reaction-separation coupling technology, the synthesis and application of nano-light (piezoelectric) catalytic materials, the preparation of hydrogen and oxygen, and the basic research of separation applications [45,46].

Notably, a prominent research collective including core authors like Toshinori Tsuru, Shaomin Liu, Weihong Xing, Nanping Xu, and Juergen Caro has exhibited the most substantial level of cooperation. Other key contributors such as Masakoto Kanezashi, Haihui Wang, and Yiqun Fan have also played significant roles in the collaborative network.

### 3.5. Research Hot Spots

**Figure 7:**
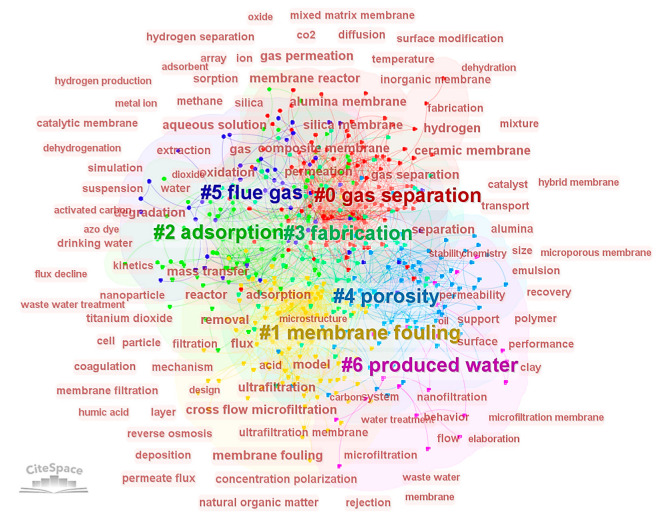
Cluster network of keywords (based on CiteSpace).

**Figure 8:**
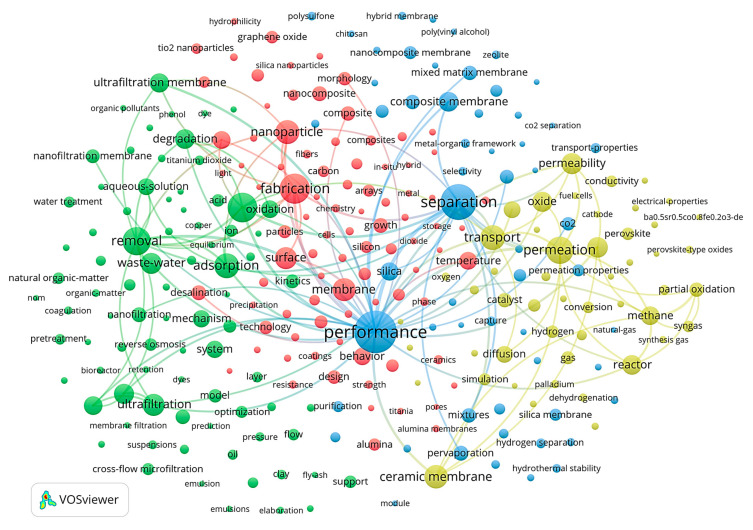
Cluster network of keywords (based on VOSviewer).

Constructing a co-occurrence map of keywords and performing clustering analysis offers insights into the prevailing research themes within the ceramic membrane field. Both CiteSpace (version: 6.2.R2) and VOSviewer (version: 1.6.16) software were utilized to cluster keywords from the accumulated literature as presented in Figure 7 and Figure 8. Keywords within the same cluster typically exhibit strong correlations and connections. Seven distinct clusters were delineated using CiteSpace (Figure 7), labeled as follows: 0# gas separation, 1# membrane fouling, 2# adsorption, 3# fabrication, 4# porosity, 5# flue gas, and 6# produced water.

In the CiteSpace results, a modularity value (*Q* value) greater than 0.3 denotes a significant clustering structure, while a Mean Silhouette (*S* value) above 0.5 indicates reasonable clustering, and above 0.7 signifies highly convincing clustering. For the keyword analysis in this study, a *Q* value of 0.46 confirms that the clustering is significant and robust. The seven clusters have an average *S* value of 0.80, suggesting high homogeneity within clusters and reliability in the clustering results. CiteSpace’s clustering of keywords reveals that gas separation, flue gas, and produced water are central to the application scenarios discussed in the literature. The central themes of gas separation and flue gas exhibit a close relationship with porosity and adsorption. Water treatment is a prominent application for ceramic membranes, with produced water and associated high-frequency terms like water treatment, wastewater, and drinking water forming part of this cluster. Membrane fouling—a common challenge in liquid treatment due to solute adsorption and concentration polarization—has a significant overlap with the cluster concerning produced water.

VOSviewer’s results (Figure 8) align with those from CiteSpace, albeit with a more extensive display of keywords and clearer delineation of inter-keyword relationships. For instance, within the green cluster, the keyword *water* is tightly linked to membrane process terms like *ultrafiltration*. Conversely, more specific terms denoting separation systems, such as *pigments*, *copper*, and *heavy metals*, are dispersed. The *fabrication* category within the red cluster shows a strong association with terms like *surface* and *nanoparticle*, underscoring the importance of membrane surface properties and their modification to meet specific requirements. This multilayered keyword analysis enables the identification of research hotspots and provides a deeper understanding of the intricate relationships between different research themes in the field of ceramic membranes.

**Figure 9:**
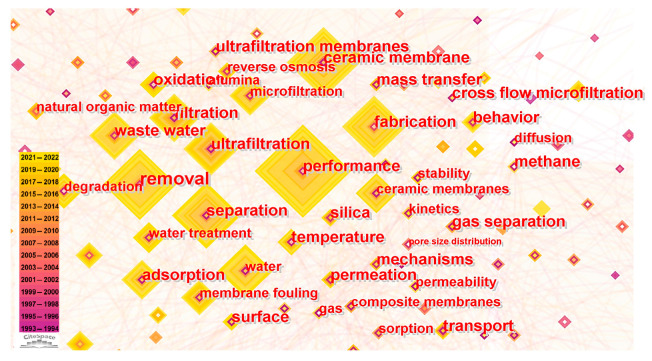
Co-occurrence map of the top keywords in the area of ceramic membranes.

The keyword co-occurrence analysis of roughly 7400 articles related to ceramic porous membranes resulted in the selection of keywords with notable frequency and centrality to underscore research foci (Figure 9). Keywords are represented as square nodes, with the node size indicating the frequency and centrality of each keyword. The color gradient surrounding each square denotes the temporal emergence of the keyword, with the outermost color representing the most recent year. After excluding generic terms like *ceramic membrane*, the remaining keywords predominantly align with three themes: Firstly, research on membrane material properties and mechanisms, such as *pore size distribution*, *diffusion*, and *mass transfer*. These terms exhibit lower centrality and, notably, have had fewer occurrences in the last five years (2017–2022). Secondly, research concerning membrane fabrication involves general methods like *fabrication*, materials such as *silica* and *alumina*, and processes including *oxidation* and *kinetics*. Thirdly, practical applications of membranes are highlighted by keywords like *performance*, *separation*, *microfiltration*, and *membrane fouling*, along with specific objects such as *water* and *wastewater*.

The temporal extent and central position of the second and third themes indicate a maturing comprehension of the relationship between materials and their applications. Researchers are progressively forming a generic strategy for designing, preparing, and utilizing ceramic membranes tailored to specific application processes. The pronounced nodes of the third theme highlight the research activities focused on the specific applications of ceramic membranes, signifying a strong movement toward their engineering and industrial uses. It is important to note that the detailed keyword distribution also shows a dispersed pattern, with some keywords approaching but not reaching a burst threshold. Therefore, a more thorough analysis is necessary to look beyond merely identifying burst keywords for fully understanding the research dynamics and directions on ceramic membranes.

**Figure 10:**
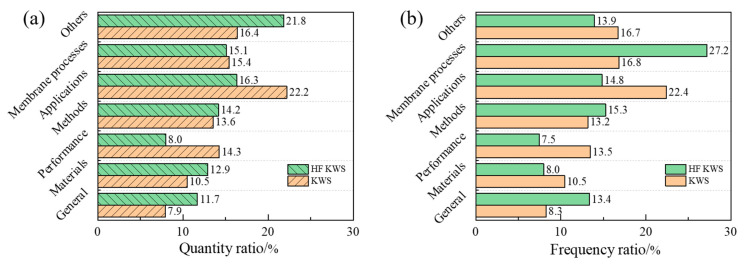
Classification and comparison of keywords. (**a**) Quantity percentage of keywords and high-frequency keywords. (**b**) Frequency percentage of keywords and high-frequency keywords.

Keywords were counted and categorized into seven groups: (1) General, such as *ceramic membrane* and *inorganic ceramic membrane*; (2) Materials, such as *alumina* and *titanium oxide*; (3) Performance, like *membrane separation* and *membrane flux*; (4) Methods, such as *sol-gel method* and phase *transformation*; (5) Applications, such as *wastewater treatment* and *Chinese medicine extraction*; (6) Membrane Processes, like *microfiltration* and *ultrafiltration*; and (7) Others. About 325 high-frequency keywords (HFKWS) with more than 30 occurrences were cataloged, totaling approximately 34,400 mentions. Additionally, around 430 keywords (KWS) with 15 to 30 occurrences were classified, amassing about 850 mentions. For about 7400 publications related to porous ceramic membranes, the results show significant variance in keyword quantity (Figure 10a) and frequency (Figure 10b) within categories. For instance, high-frequency keywords in the *Materials* category represent about 12.9% in quantity but 8.0% in frequency, reflecting a dispersed frequency distribution and often smaller counts per keyword. This is mainly due to those keywords with overlapping meanings or inclusion relationships, like *silica*, *SiO_2_*, and *silicon*, often being used differently by different authors. This is also true for the *Methods* category, where terms like *sol*, *gel*, and *sol-gel* may reflect similar processes but are labeled differently. Conversely, high-frequency keywords related to *Membrane Processes* have a frequency ratio significantly outweighing their quantity ratio, indicating that the expression on membrane processes is relatively concentrated by different authors.

In addition, during the comparison process, it was also found that there are large differences in the statistical results of high-frequency keywords and keywords. For example, the total frequency of *Applications* category high-frequency keywords is only 14.8%, markedly less than the 22.4% of keywords. This could be due to the diversity of application scenarios beyond water treatment, leading to a less focused keyword concentration. The *Performance* category mirrors that of the *Applications* category, mainly because performance is closely related to application scenarios. This exemplifies a long-tail effect in ceramic membrane applications research [47]: a diverse array of application scenarios, each with low-frequency feature keywords but collectively contributing to a large total number. This suggests that the long tail of ceramic membrane applications harbors many potential research areas and emerging points that warrant further exploration and application.

### 3.6. High-Impact Publications

Publications with a high citation count are typically indicative of significant academic value and professional impact. Citation burst detection is sometimes used to identify periods of substantial change in citation activity, pinpointing when a particular work has garnered significant attention. To effectively emphasize the literature during burst detection, it necessitates not only a high citation number but also a frequency surpassing the average within the same period. This approach differs from citation statistics alone and provides a way to find the emergence and evolution of research hotspots over time.

**Figure 11:**
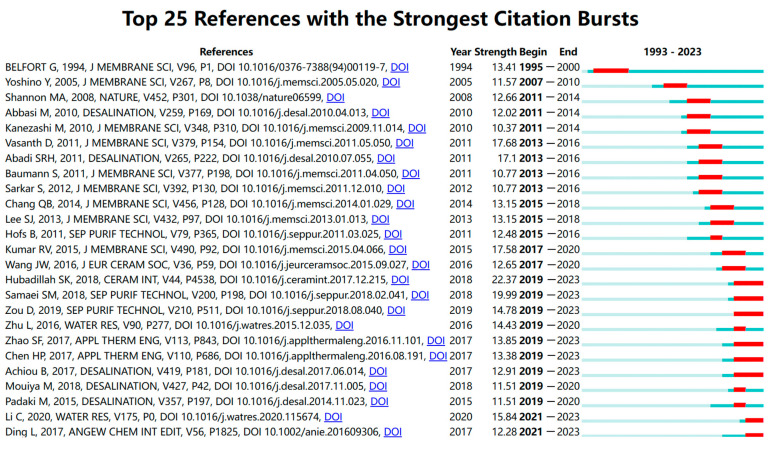
Citation bursts of highly cited publications.

**Figure 12:**
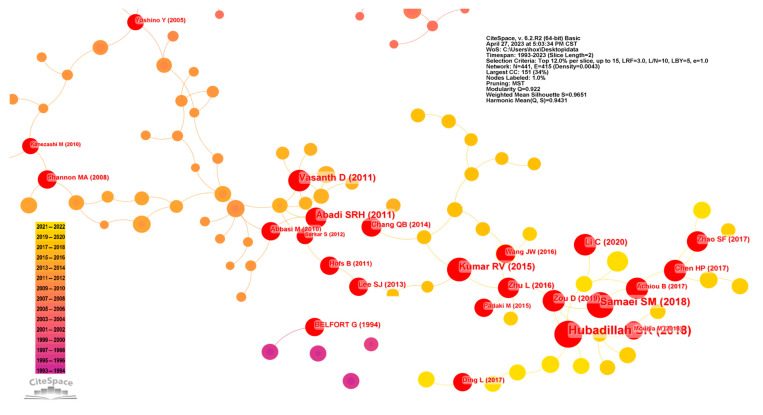
Co-occurrence map of highly cited publications.

In the analysis of approximately 7400 publications concerning porous ceramic membranes, both co-occurrence mapping and citation burst detection were conducted (Figure 11 and Figure 12), accounting for the lag between publication and citation with the latest detection year set to 2020. This methodology led to the identification of around 20 highly cited publications.

The emergence of burst citations commenced in 2008, aligning with the publication growth trend identified in Section 3.2: notably, the surge in publications around 2009. Prior to 2008, research primarily concentrated on the integration of membrane processes and the design and fabrication of membrane elements and modules. Studies subsequent to this period shifted focus toward performance evaluations within specific applications. Among the eight highly cited publications spanning 2008 to 2015, five publications explored membrane performance in particular applications, with a notable emphasis on water treatment and related separation for liquid systems. The remaining three publications addressed the cost-effectiveness of membranes, including the utilization of natural minerals for cost-effective production and solving fouling issues to enhance membrane lifespan.

From 2015 to 2020, ten significant publications were identified, predominantly addressing the cost aspects of separation processes from varied perspectives. There was a noticeable surge in research aimed at reducing production costs by utilizing low-cost materials like clay, volcanic ash, and fly ash for the fabrication of membranes. Moreover, there was a focus on decreasing operational energy consumption through the exploration of process intensification techniques, such as employing membrane condensers to enhance heat exchange efficiency in stripping processes. In contrast to earlier research, recent studies often target more specialized applications, increasingly factoring in the engineering and practical application potential of membranes alongside their economic feasibility. This evolution indicates a broader trend in the field toward the development of separation technologies that are not only more sustainable but also economically viable. In summary, the findings indicate that the research emphasis predominantly revolves around the fabrication of ceramic membranes and their performance in various applications. These insights corroborate the result identified from the keyword co-occurrence analysis in Section 3.5.

## 4. Conclusions

Taking ceramic membranes as the object, this article conducts a quantitative and visual analysis of the literature based on the WOS database with the help of CiteSpace (version: 6.2.R2) and VOSviewer (version: 1.6.16) software. The following conclusions are obtained:

(1) A retrieval strategy and a formula for ceramic membrane literature were established, yielding approximately 15,250 refined articles. The retrieval approach was assessed on recall and precision rates, achieving 84.20% and 74.33%, respectively, laying a solid foundation for further bibliometric and visual analysis.
(2) An examination of the literature distribution across geographical, temporal, and thematic dimensions reveals that the research on ceramic membranes has reached a stage of maturity. Significantly, the contributions from China, the USA, Germany, and Japan are predominant in this field, with China alone contributing over half of the total publications by 2022. The top ten journals collectively constitute 29.81% of the publications, reflecting a concentrated yet varied landscape of research primarily situated within the domains of Chemical Engineering and Water Resources.
(3) The employment of visual analytics tools like VOSviewer and CiteSpace has facilitated the delineation of author co-occurrence networks and the clustering of keywords. This analysis has revealed a tightly knit core group of authors and has uncovered the long-tail effect within the realm of ceramic membrane applications. This long tail signifies a spectrum of numerous potential research areas and emerging topics within ceramic membranes that are ripe for further exploration and practical application.
(4) CiteSpace’s burst detection feature has uncovered the key literature on porous ceramic membranes that corresponds with significant periods of development in this research field. Burst activity, beginning around 1995 and becoming more pronounced around 2011, indicates a shift in research dynamics. Initial studies were primarily focused on membrane process design, material development, and fundamental properties. More recent research, however, has pivoted towards optimizing cost-efficiency and enhancing the performance of membranes within specific applications, indicating a trend toward practical implementation and industry-oriented solutions.

## Figures and Tables

**Table 1:** Retrieval strategy evaluation statistics.

Institutions	*N*_P_	*N*_A_
Chinese Academy of Sciences	930	1107
Centre National de la Recherche Scientifique (CNRS)	623	745
Nanjing Tech University	530	561
UDICE-French Research Universities	300	424
Helmholtz Association	260	302
Total	2643	3139

Note: The time range is from 1 January 1990 to 30 December 2022. Among them, *N*_A_ is the total number of papers published by the organization in the field of ceramic membrane, *N*_P_ is the number of articles by the organization in the retrieval results.

**Table 2:** Annual publications and citations.

Years	Publications	Citations	Ratio/%
1992	84	139	1.65
1996	151	896	5.75
2000	299	2626	8.78
2004	391	5446	13.93
2008	510	10,843	21.26
2012	655	18,621	28.43
2016	1033	29,973	29.02
2020	1311	53,455	40.77

**Table 3:** The number of publications, patents, and projects on ceramic membranes from China.

Years	Publications from CNKI	Patents from China	Projects from NSFC
1990	0	1	1
1991	1	2	1
1992	0	1	3
1993	1	0	4
1994	6	2	1
1995	6	4	8
1996	3	5	2
1997	14	11	2
1998	19	7	2
1999	26	13	4
2000	41	19	2
2001	58	27	5
2002	42	25	5
2003	55	36	2
2004	58	50	9
2005	53	74	9
2006	86	111	8
2007	58	116	10
2008	80	188	8
2009	93	221	16
2010	88	228	16
2011	95	328	18
2012	77	449	20
2013	94	516	19
2014	115	634	23
2015	155	874	27
2016	168	989	20
2017	173	1191	21
2018	174	1543	20
2019	210	1605	
2020	189	1664	
2021	219	1968	
2022	170	1899	

**Table 4:** The top 10 journals for ceramic membrane publications.

Rank	Journals	Publications	Categories
1	Journal of Membrane Science	2021	Engineering, Chemical Polymer Science
2	Separation and Purification Technology	808	Engineering, Chemical
3	Desalination	637	Engineering, Chemical Water Resources
4	Desalination and Water treatment	389	Engineering, Chemical Water Resources
5	Industrial Engineering Chemistry Research	351	Engineering, Chemical
6	Chemical Engineering Journal	390	Engineering, Chemical Engineering, Environmental
7	Ceramics International	270	Materials Science, Ceramics
8	International Journal of Hydrogen Energy	257	Chemistry, Physical Electrochemistry Energy and Fuels
9	RSC Advances	227	Chemistry, Multidisciplinary Chemistry, Physical Materials Science, Multidisciplinary
10	ACS Applied Materials Interfaces	207	Materials Science, Multidisciplinary Nanoscience and Nanotechnology

## Data Availability

Not applicable.

## Supplementary Materials

The following are available online at https://www.mdpi.com/article/10.3390/membranes14070144/s1, Table S1: Publications and citations on ceramic membranes in different journals.

## References

[1] Mao X., Wang Y., Yan X., Huang Z., Gao Z., Wang Y., Huang L., Kipper M.J., Tang J. (2023). A review of superwetting membranes and nanofibers for efficient oil/water separation. J. Mater. Sci..

[2] Chrisma R.B., Jafri R.I., Anila E.I. (2023). A review on the electrochemical behavior of graphene–transition metal oxide nanocomposites for energy storage applications. J. Mater. Sci..

[3] Sazali N. (2020). A review of the application of carbon-based membranes to hydrogen separation. J. Mater. Sci..

[4] Elimelech M., Phillip W.A. (2011). The future of seawater desalination: Energy, technology, and the environment. Science.

[5] Guliants V.V., Carreon M.A., Lin Y.S. (2004). Ordered mesoporous and macroporous inorganic films and membranes. J. Membr. Sci..

[6] Ovalle-Encinia O., Lin J.Y.S. (2023). Synthesis and characteristics of porous ceramic tubes: A comparison of centrifugal casting and cold isostatic pressing methods. J. Mater. Sci..

[7] Asif M.B., Zhang Z.H. (2021). Ceramic membrane technology for water and wastewater treatment: A critical review of performance, full-scale applications, membrane fouling and prospects. Chem. Eng. J..

[8] Gao N., Fan Y., Quan X., Cai Y., Zhou D. (2016). Modified ceramic membranes for low fouling separation of water-in-oil emulsions. J. Mater. Sci..

[9] Samaei S.M., Gato-Trinidad S., Altaee A. (2018). The application of pressure-driven ceramic membrane technology for the treatment of industrial wastewaters—A review. Sep. Purif. Technol..

[10] Das B., Chakrabarty B., Barkakati P. (2016). Preparation and characterization of novel ceramic membranes for micro-filtration applications. Ceram. Int..

[11] Wang H., Cai Z., Li S., Zheng J., Xie Y., He Y., Li C., Zheng D. (2023). Research hotspots and frontiers of post-stroke aphasia rehabilitation: A bibliometric study and visualization analysis. Front. Hum. Neurosci..

[12] Xue J., Han R., Li Y., Zhang J., Liu J., Yang Y. (2023). Advances in multiple reinforcement strategies and applications for silica aerogel. J. Mater. Sci..

[13] Klavans R., Boyack K.W. (2006). Identifying a better measure of relatedness for mapping science. J. Am. Soc. Inf. Sci. Technol..

[14] Zhang L., Zhong Y., Geng Y. (2019). A bibliometric and visual study on urban mining. J. Clean. Prod..

[15] Ellegaard O. (2018). The application of bibliometric analysis: Disciplinary and user aspects. Scientometrics.

[16] Ferreira J.J.M., Ferreira F.A.F., Fernandes C.I.M.A.S., Jalali M.S., Raposo M.L., Marques C.S. (2016). What do we not know about technology entrepreneurship research?. Int. Entrep. Manag. J..

[17] Ang W.L., Mohammad A.W., Johnson D., Hilal N. (2019). Forward osmosis research trends in desalination and wastewater treatment: A review of research trends over the past decade. J. Water Process Eng..

[18] Escobar Yonoff R., Ochoa G.V., Cardenas-Escorcia Y., Ivan Silva-Ortega J., Merino-Stand L. (2019). Research trends in proton exchange membrane fuel cells during 2008-2018: A bibliometric analysis. Heliyon.

[19] Wang C.-C., Ho Y.-S. (2016). Research trend of metal-organic frameworks: A bibliometric analysis. Scientometrics.

[20] Naseer M.N., Dutta K., Zaidi A.A., Asif M., Alqahtany A., Aldossary N.A., Jamil R., Alyami S.H., Jaafar J. (2022). Research trends in the use of polyaniline membrane for water treatment applications: A scientometric analysis. Membranes.

[21] Miranda D.M.V.d., Dutra L.d.S., Way D., Amaral N., Wegenast F., Scaldaferri M.C., Jesus N., Pinto J.C. (2019). A bibliometric survey of paraffin/olefin separation using membranes. Membranes.

[22] Aytaç E., Khayet M. (2023). A topic modeling approach to discover the global and local subjects in membrane distillation separation process. Separations.

[23] Li W., Dong H., Yu H., Wang D., Yu H. (2018). Global characteristics and trends of research on ceramic membranes from 1998 to 2016: Based on bibliometric analysis combined with information visualization analysis. Ceram. Int..

[24] Kwok K.L. (1995). A network approach to probabilistic information-retrieval. ACM Trans. Inf. Syst..

[25] Ye C., Sun H.-J., Xu Q., Liang T., Zhang Y., Liu Q. (2019). Working memory capacity affects trade-off between quality and quantity only when stimulus exposure duration is sufficient: Evidence for the two-phase model. Sci. Rep..

[26] Chen C.M., Hu Z.G., Liu S.B., Tseng H. (2012). Emerging trends in regenerative medicine: A scientometric analysis in CiteSpace. Expert Opin. Biol. Ther..

[27] Gehanno J.F., Thaon I., Pelissier C., Rollin L. (2023). Precision and recall of search strategies for identifying studies on work-related psychosocial risk factors in PubMed. J. Occup. Rehabil..

[28] Ding X., Yang Z. (2022). Knowledge mapping of platform research: A visual analysis using VOSviewer and CiteSpace. Electron. Commer. Res..

[29] Chen C.M. (2006). CiteSpace II: Detecting and visualizing emerging trends and transient patterns in scientific literature. J. Am. Soc. Inf. Sci. Technol..

[30] van Eck N.J., Waltman L. (2010). Software survey: VOSviewer, a computer program for bibliometric mapping. Scientometrics.

[31] Woolston C. (2021). Scientists count the career costs of COVID. Nature.

[32] Mongeon P., Paul-Hus A. (2016). The journal coverage of Web of Science and Scopus: A comparative analysis. Scientometrics.

[33] Wang Y.F., Yan C.G., Xiao Z.H., Luo X.N. (2020). Technological Innovation Trend of Ceramic Membranes: A Study Based on the Patent Information Analysis. J. Ceram..

[34] Feng J., Ke W., Qiu H. (2021). Research on the development of waste oil treatment using membrane separation technology from the perspective of patent. Membr. Sci. Technol..

[35] Shneider A.M. (2009). Four stages of a scientific discipline; four types of scientist. Trends Biochem. Sci..

[36] Zaviska F., Drogui P., Grasmick A., Azais A., Héran M. (2013). Nanofiltration membrane bioreactor for removing pharmaceutical compounds. J. Membr. Sci..

[37] George S.M., Kandasubramanian B. (2020). Advancements in MXene-Polymer composites for various biomedical applications. Ceram. Int..

[38] Zhang D., Kanezashi M., Tsuru T., Yamamoto K., Gunji T., Adachi Y., Ohshita J. (2023). Preparation of thermally stable 3-glycidyloxypropyl-POSS-derived polysilsesquioxane RO membranes for water desalination. J. Membr. Sci..

[39] Moriyama N., Nagasawa H., Kanezashi M., Tsuru T. (2022). Water permeation in gas and liquid phases through organosilica membranes: A unified theory of reverse osmosis, pervaporation, and vapor permeation. Chem. Eng. Sci..

[40] Fan H.W., Wang H.R., Peng M.H., Meng H., Mundstock A., Knebel A., Caro J. (2023). Pore-in-Pore Engineering in a Covalent Organic Framework Membrane for Gas Separation. ACS Nano.

[41] Li Z.K., Liu Y.C., Li L.B., Wei Y.Y., Caro J., Wang H.H. (2019). Ultra-thin titanium carbide (MXene) sheet membranes for high-efficient oil/water emulsions separation. J. Membr. Sci..

[42] Chen P., Ma X., Zhong Z., Zhang F., Xing W., Fan Y. (2017). Performance of ceramic nanofiltration membrane for desalination of dye solutions containing NaCl and Na_2_SO_4_. Desalination.

[43] Wei W., Zhang W., Jiang Q., Xu P., Zhong Z., Zhang F., Xing W. (2017). Preparation of non-oxide SiC membrane for gas purification by spray coating. J. Membr. Sci..

[44] Yang Y., Xu W., Zhang F., Low Z.-X., Zhong Z., Xing W. (2017). Preparation of highly stable porous SiC membrane supports with enhanced air purification performance by recycling NaA zeolite residue. J. Membr. Sci..

[45] Hei Y.P., Wu S., Lu Z.J., Meng X., Song J., Yang N., Meng B., Li C., Sunarso J., Kawi S. (2024). Dual-phase Ce_0.8_Sm_0.2_O_2−δ_-La_0.8_Ca_0.2_Al_0.3_Fe_0.7_O_3−δ_ oxygen permeation hollow fiber membrane for oxy-CO_2_ reforming of methane. Catal. Sci. Technol..

[46] Chen G.X., Widenmeyer M., Yu X., Han N., Tan X.Y., Homm G., Liu S.M., Weidenkaff A. (2024). Perspectives on achievements and challenges of oxygen transport dual-functional membrane reactors. J. Am. Ceram. Soc..

[47] Ruocco G., Daraio C., Folli V., Folli V. (2017). Bibliometric indicators: The origin of their log-normal distribution and why they are not a reliable proxy for an individual scholar’s talent. Palgrave Commun..

