# Supplementary material for: Bibliometric and Visual Analysis of Studies on Ceramic Membranes: A Review
Source: Membranes (Basel). 2024 Jun 25;14(7):144. doi: 10.3390/membranes14070144 (PMC11278809; doi:10.3390/membranes14070144)
Supplement: Supplementary file 1 [file membranes-14-00144-s001.zip › membranes-3056712-supplementary.pdf]

Table S1. Publications and citations on ceramic membranes in different journals.

Journals	Publications	Citations	Total Link Strength
Journal of Membrane Science	2021	91808	22777
Separation and Purification Technology	808	23113	8723
Desalination	637	23968	5143
Desalination and Water Treatment	389	2749	2257
Industrial & Engineering Chemistry Research	351	9720	3751
Chemical Engineering Journal	347	11436	3088
Ceramics International	270	4418	3117
International Journal of Hydrogen Energy	257	6233	1896
RSC Advances	227	4030	1362
ACS Applied Materials & Interfaces	207	7557	1296
Catalysis Today	187	7122	2275
Journal of the European Ceramic Society	182	4593	2536
Microporous and Mesoporous Materials	176	4957	1879
Water Research	169	8792	1489
Separation Science and Technology	164	3450	1264
Langmuir	161	5710	392
Solid State Ionics	147	7846	2080
Journal of Applied Polymer Science	145	2574	736
Journal of Colloid and Interface Science	143	5338	1029
AICHE Journal	138	4877	2844
Applied Physics Letters	130	6049	121
Environmental Science & Technology	125	7728	801
Journal of Hazardous Materials	121	5038	801
Applied Surface Science	119	4428	895
Chemical Engineering Science	117	4301	1464
Journal of Materials Chemistry A	114	4307	1285
Nanotechnology	112	2107	176

Colloids and Surfaces A-physicochemical and Engineering Sspects	111	2104	548
Chemosphere	104	1877	528
Journal of Water Process Engineering	103	1298	845
Chemical Engineering Research & Design	101	2380	871
Journal of Materials Science	101	1610	612
Water Science and Technology	101	1415	347
Journal of Micromechanics and Microengineering	100	1924	75
Journal of Environmental Chemical Engineering	96	944	710
Nano Letters	95	9023	274
Journal of Vacuum Science & Technology B	94	1581	36
Sensors and Actuators A-Physical	87	2142	42
Materials Letters	82	1390	468
Journal of Physical Chemistry C	81	2427	370
Scientific Reports	81	2273	206
Journal of the American Ceramic Society	79	2050	982
Korean Journal of Chemical Engineering	78	955	553
Journal of the Electrochemical Society	73	2790	511
Journal of Inorganic Materials	67	193	279
Chemical Engineering and Processing-Process Intensification	66	1340	478
ACS Nano	63	4292	273
Journal of Applied Physics	61	1153	62
Journal of Nanoscience and Nanotechnology	61	510	136
Analytical Chemistry	60	2546	40
Chinese Journal of Chemical Engineering	60	710	614
Journal of Cleaner Production	60	1407	372
Journal of Microelectromechanical Systems	60	1814	60
Journal of Power Sources	60	2477	175
Chemical Communications	59	2603	795

Journal of Industrial and Engineering Chemistry	59	1138	488
Polymers	59	378	263
Applied Catalysis A-General	58	2267	636
Chemistry of Materials	58	4494	807
Applied Catalysis B-Environmental	57	2605	354
Environmental Science and Pollution Research	57	584	282
Advanced Materials	56	4081	596
Journal of Materials Chemistry	56	3065	383
Journal of Physical Chemistry B	56	2196	164
Nanoscale	56	1871	172
Journal of Alloys and Compounds	52	932	456
Journal of Sol-Gel Science and Technology	52	815	404
Science of the Total Environment	50	1194	170
Electrochimica Acta	49	1469	80
Physical Chemistry Chemical Physics	47	1519	205
Chemical Engineering & Technology	46	345	302
Journal of the Taiwan Institute of Chemical Engineers	46	787	336
Applied Clay Science	45	1401	419
Materials	45	353	229
Energy & Fuels	44	929	296
Journal of Chemical Engineering of Japan	44	654	463
Environmental Technology	43	430	187
Nanomaterials	43	501	184
Polymer	43	2005	288
Chemistry Letters	42	422	131
ACS Sustainable Chemistry & Engineering	41	1138	220
Advanced Functional Materials	41	2832	403
Materials Chemistry and Physics	41	596	237
Microsystem Technologies: Micro- and	41	436	8

Nanosystems Information Storage and Processing Systems			
Analytica Chimica Acta	38	1384	35
Angewandte Chemie-International Edition	38	1967	298
ACS Applied Nano Materials	37	338	168
Chemical Journal of Chinese Universities-Chinese	37	147	87
Physical Review B	37	1624	66
Journal of Porous Materials	36	595	249
Small	36	1628	86
Journal of environmental management	35	587	266
Journal of the American Chemical Society	35	4380	308
Journal of the Ceramic Society of Japan	33	287	245
Nuclear Instruments & Methods in Physics Research Section B-Beam	33	448	18
New Journal of Chemistry	32	263	130
Review of Scientific Instruments	32	743	25
Journal of Water Chemistry and Technology	31	73	73
Acta Chimica Sinica	30	107	47
Carbon	30	1097	145
Catalysis Letters	30	956	379
Talanta	30	988	33
